# The expression of Dicer and Drosha in matched normal tissues, tumours and lymph node metastases in triple negative breast cancer

**DOI:** 10.1186/1471-2407-14-253

**Published:** 2014-04-11

**Authors:** Kelly A Avery-Kiejda, Stephen G Braye, John F Forbes, Rodney J Scott

**Affiliations:** 1 Centre for Information-Based Medicine, Hunter Medical Research Institute, John Hunter Hospital, New Lambton Heights, NSW 2305, Australia; 2 School of Biomedical Sciences and Pharmacy, Faculty of Health, University of Newcastle, Callaghan, NSW 2308, Australia; 3 Hunter Area Pathology Service, John Hunter Hospital, New Lambton Heights, NSW 2305, Australia; 4 Australian New Zealand Breast Cancer Trials Group and, Department of Surgical Oncology, Calvary Mater Newcastle Hospital, Waratah, NSW 2298, Australia; 5 School of Medicine and Public Health, Faculty of Health, University of Newcastle, Callaghan, NSW 2308, Australia

**Keywords:** Dicer, Drosha, Breast cancer, Metastasis, Triple negative

## Abstract

**Background:**

Breast cancer is the most common malignancy in women world-wide. Triple negative breast cancer (TNBC) is a highly aggressive subtype that lacks expression of hormone receptors for estrogen, progesterone and human epidermal growth factor 2; and is associated with a high propensity for metastatic spread. Several studies have identified critical roles for microRNAs in breast cancer, but the role of two critical enzymes involved in microRNA biogenesis, Dicer and Drosha, is not well understood, particularly with respect to metastatic progression in this subtype.

**Methods:**

We examined the expression of Dicer and Drosha in a series of invasive 35 TNBCs with matched normal adjacent tissues (n = 18) and lymph node metastases (n = 15) using semi-quantitative real time RT-PCR. The relationship of their expression with clinical features including age at diagnosis, lymph node positivity and tumour size was analysed.

**Results:**

We report that Dicer was significantly decreased while Drosha was significantly increased in tumours when compared to normal adjacent tissues. While there was no difference in Drosha expression in lymph node metastases when compared to the primary tumour, Dicer was significantly increased. There was no correlation between the expression of either Dicer or Drosha to age at diagnosis, lymph node positivity and tumour size.

**Conclusions:**

In conclusion, Dicer and Drosha are dysregulated in TNBC and matched lymph node metastases however, the clinical relevance of this is still not known. The altered expression of Dicer and Drosha may serve as markers for disrupted miRNA biogenesis in TNBC.

## Background

Breast cancer is the most common malignancy that develops in women worldwide, responsible for the highest cancer-associated death rates [1]. Triple negative breast cancer (TNBC) represents an important clinical subtype, characterised by an absence of estrogen receptor (ER), progesterone receptor (PR) and human epidermal growth factor receptor 2 (HER2) and which therefore lack common targets used for anti-hormone therapies [2,3]. Although TNBCs comprise only a small percentage of all breast cancers diagnosed (10-24%), patients are of younger age, tend to develop tumours of larger size, and have an increased likelihood of distant metastasis and death within 5 years of diagnosis [2,3]. Thus, TNBCs represent a major problem for which targeted therapies are currently not available.

microRNAs (miRNAs) are a class of small (~22 nucleotides) non-coding RNAs that control gene expression by targeting mRNAs and triggering either translational repression or RNA degradation [4]. Several studies have identified critical roles for miRNAs in breast cancer diagnosis and prognosis [5-10]. Two enzymes, Drosha and Dicer, are pivotal in the processing of pri-miRNA into mature double stranded miRNA fragments [4]. Drosha is a nuclear enzyme that cleaves primary miRNA transcripts (pri-miRNA) into short (~70 nucleotides) double-stranded RNA precursors that contain a 3’ overhang, known as pre-miRNA [11]. The pre-miRNAs are then exported to the cytoplasm where they are cleaved by Dicer into mature double-stranded miRNA fragments of approximately 22 nucleotides in length [11]. Numerous studies have investigated the role of Drosha and Dicer in a variety of cancers including breast, lung, ovarian, colorectal and esophageal cancer [12-23]. In breast cancer, both Dicer and Drosha expression have been reported to be reduced when compared to normal adjacent tissue [18-23]. Reduced expression of Dicer has been associated with high grade, shorter metastasis-free survival and with the TNBC subtype [19,21-23]. A reduction in the expression of Drosha in breast cancer has been reported to be associated with high grade and shorter disease free survival [18,21]. However, there have been no studies regarding the association of Dicer or Drosha with clinical features or their role in breast cancer progression in TNBC, a highly aggressive breast cancer subtype with a propensity for metastatic spread.

In this study we examined the expression of Dicer and Drosha in a series of 35 TNBCs with matched normal adjacent tissues (n = 18) and lymph node (LN) metastases (n = 15) using semi-quantitative real time RT-PCR. The correlation of their expression with clinical features including age at diagnosis, LN positivity and tumour size was also examined.

## Methods

### Study cohort

Thirty-five formalin-fixed paraffin-embedded (FFPE) invasive ductal carcinomas (IDCs) were obtained from Hunter Area Pathology Service, John Hunter Hospital, Newcastle, Australia. All patients were diagnosed with grade 3 IDC between the years of 2004-2009, and were negative for ER, PR and HER2 as assessed through routine diagnostic pathology. The demographic details of this cohort have previously been published [24]. Areas of tissue representing histologically normal adjacent breast tissue (NAT, where available, n = 18), IDC and LN metastases (n = 15) were identified and confirmed by a pathologist. Micrometastases (<2 mm) were not used. A 1.5 mm punch biopsy was used to punch cores from the paraffin blocks using haemotoxylin and eosin stained sections of the same sample for guidance. Tumour volume in the core biopsy was >70% of the total. This study complies with the Helsinki Declaration with ethical approval from the Hunter New England Human Research Ethics Committee (Approval number: 09/05/20/5.02). In accordance with the *National Statement on Ethical Conduct in Research Involving Humans*, a waiver of consent was granted for this study.

### Extraction of RNA

Total RNA was extracted using the miRNeasy FFPE kit (Qiagen, Doncaster, VIC, Australia). RNA was quantified using the Quant-it RiboGreen RNA Assay kit (Invitrogen, Mulgrave, VIC, Australia) and purity assessed by A_260/A280_ and A_260/230_ ratios (>1.8) using the Nanodrop. The RNA integrity of selected samples was analysed using the 2100 Bioanalyser and the RNA 6000 Nano kit (Agilent Technologies, Mulgrave, VIC, Australia).

### Semi-quantitative real-time PCR

Total RNA (250 ng) was reverse transcribed using the High Capacity cDNA Reverse Transcription Kit (Life Technologies, Mulgrave, VIC, Australia) which utilises random hexamers in the reverse transcription reaction. Real-time PCR analysis was performed in triplicate using TaqMan® Universal PCR mix (Life Technologies) according to the manufacturers’ instructions, with results quantified on a 7500 real-time PCR system (Life Technologies) as described previously [25]. The expression of Dicer (Hs00998588_g1), Drosha (Hs00203008_m1) and β2-Microglobulin (Hs99999907_m1) were quantified using Taqman Gene Expression Assays (Life Technologies). The relative expression of the Dicer and Drosha were normalised to β2-Microglobulin (ΔCt) and expressed as the fold change as described previously [25]. We have verified that β2-Microglobulin is equally expressed among the different tissues analysed in this study (NAT, IDC, LN) (Additional file 1: Table S1 and Additional file 1: Figure S1).

### Statistical analysis

The normality of the data distribution was tested using a D’Agostino and Pearson Omnibus test. The values were found not to have been sampled from a Gaussian distribution and thus, non-parametric statistical tests were used to compare the data. A two-tailed Mann-Whitney U test was used to determine if there was a statistically significant difference in the expression of Dicer and Drosha between any two subgroups. The Wilcoxon matched-pairs signed rank test was used to determine if there was a statistically significant difference in the expression of Dicer and Drosha between matched pairs. The Kruskal-Wallis rank test followed by a Dunn’s Multiple Correction test was used to determine the statistical significance of Dicer and Drosha expression between multiple (>2) subgroups. Analysis of the correlation between Dicer and Drosha expression and clinical parameters was performed using Spearman’s correlation test. All analysis was performed using GraphPad Prism (version 5.04, GraphPad software Inc., La Jolla, CA, USA).

## Results

### Drosha is more highly expressed than Dicer in TNBC

**Figure 1:**
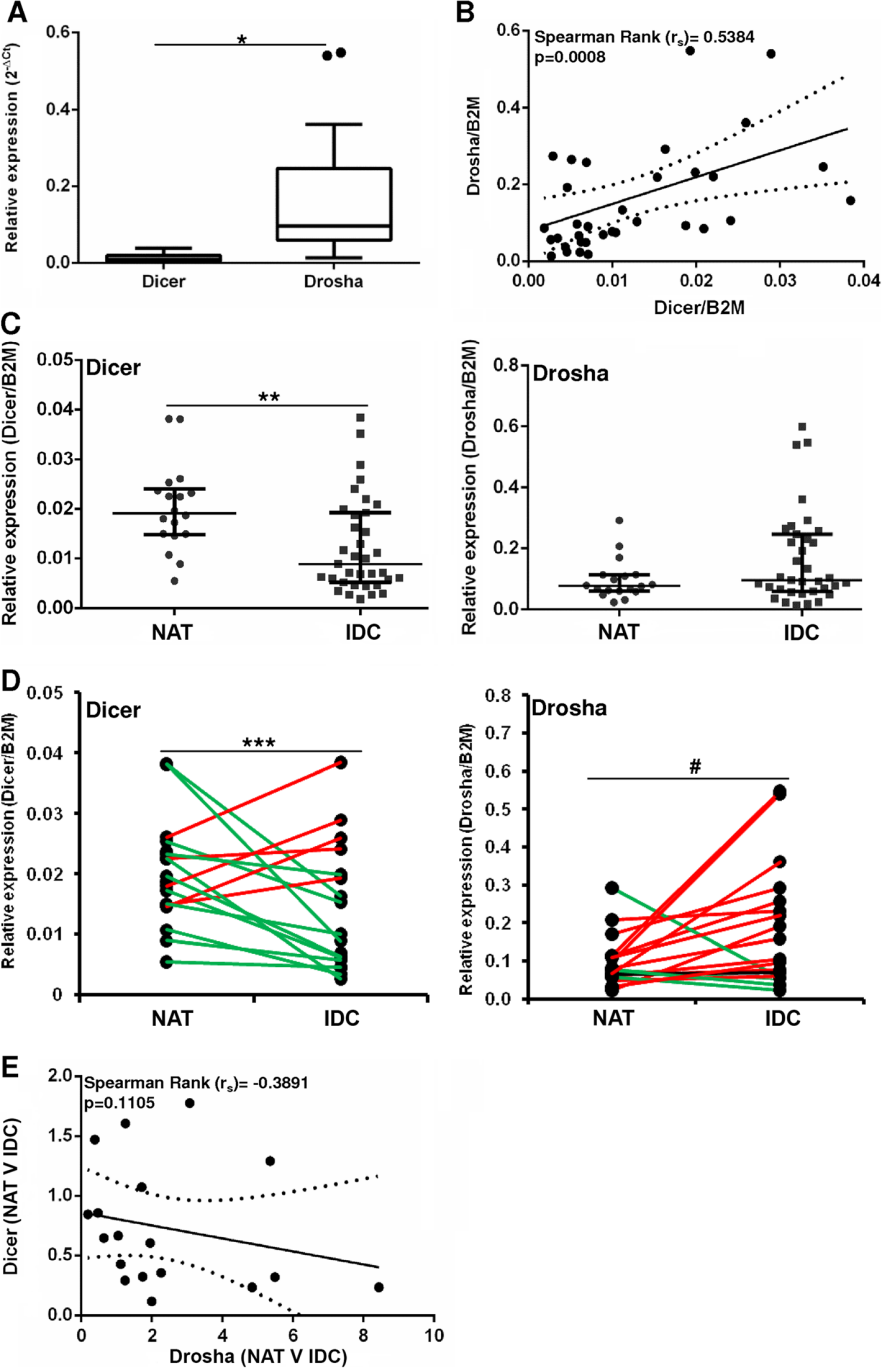
**Dicer and Drosha expression in triple negative breast cancer. A)** Relative quantification of Dicer and Drosha by real-time RT-PCR in tumour (n = 35) samples. **B)** The correlation between Dicer and Drosha expression levels. **C)** Relative quantification of Dicer and Drosha by real-time RT-PCR in all NAT (n = 18) and IDC (n = 35) samples. **D)** Relative quantification of Dicer and Drosha by real-time RT-PCR in matched NAT (n = 18) and IDC (n = 18) samples. **E)** Correlation between Dicer and Drosha fold change in IDC versus NAT samples. Results are shown as the relative normalised expression (target/β2-Microglobulin) of the target (2^-ΔCt^). Boxes **(A)** represent the median ± interquartile range. Horizontal line **(C)** represents the median ± interquartile range. Decreased and increased expression in tumour versus normal **(D)** is shown in green and red respectively. *p < 0.0001, **p = 0.0039, ***p = 0.0432, ^#^p = 0.0235.

The expression of Dicer and Drosha was quantitated in all 35 TNBCs by real time RT-PCR. The relative mRNA expression of Drosha was significantly higher (~11 fold, p < 0.0001) than the expression of Dicer (Figure 1A). In addition, the expression of Dicer and Drosha were found to be highly correlated in tumours (Figure 1B, r_s_ = 0.5384, p = 0.0008). This is consistent with the findings of Passon *et al*. and Dedes *et al.*, who also found Dicer and Drosha expression to be correlated in breast cancer [21,23].

### Dicer is decreased while Drosha is increased in TNBC when compared to matched normal tissue

There have only been two studies that have examined the relative expression of both Dicer and Drosha in TNBC in relation to the normal breast [21,23]. However, they compared their tumour specimens to a small number of unmatched normal breast tissues (n = 6, n = 10) that were derived from reduction mammoplasty. Contrasting results were found with Passon *et al.* concluding that there was no significant difference in the expression of these two genes in the normal breast compared to TNBC [23] and Dedes *et al.*, found a significant down-regulation for Dicer only [21]. Given the heterogeneous nature of the breast, we examined Dicer and Drosha expression in TNBC compared to matched normal adjacent tissues (NAT). Initially, we examined all NAT (n = 18) compared to all tumours (n = 35) and found the expression of Dicer and Drosha was highly variable in both tissue types (Figure 1C). Dicer expression was found to be significantly reduced in tumour tissue compared to NAT in the unmatched analysis (p = 0.0039), while no difference was observed for Drosha expression (Figure 1C). This is consistent with the results of Dedes *et al.*[21].

We next examined the expression of Dicer and Drosha in the matched NAT-tumour pairs (n = 18). Again, Dicer was found to be significantly reduced in 13/18 (72%) tumours compared to NAT (Figure 1D, p = 0.0432). In contrast, Drosha was significantly increased in 14/18 (78%) tumours when compared to matched NAT (Figure 1D, p = 0.0235). Given the contrasting results, we hypothesised that decreased Dicer expression may be compensating for increased Drosha expression in these tissues and examined whether the fold change in Dicer and Drosha expression in tumour versus NAT was correlated. We found no correlation between Dicer and Drosha fold induction in tumour compared to NAT (Figure 1E, p = 0.1105).

### Dicer and Drosha are not associated with clinical features of TNBC

**Figure 2:**
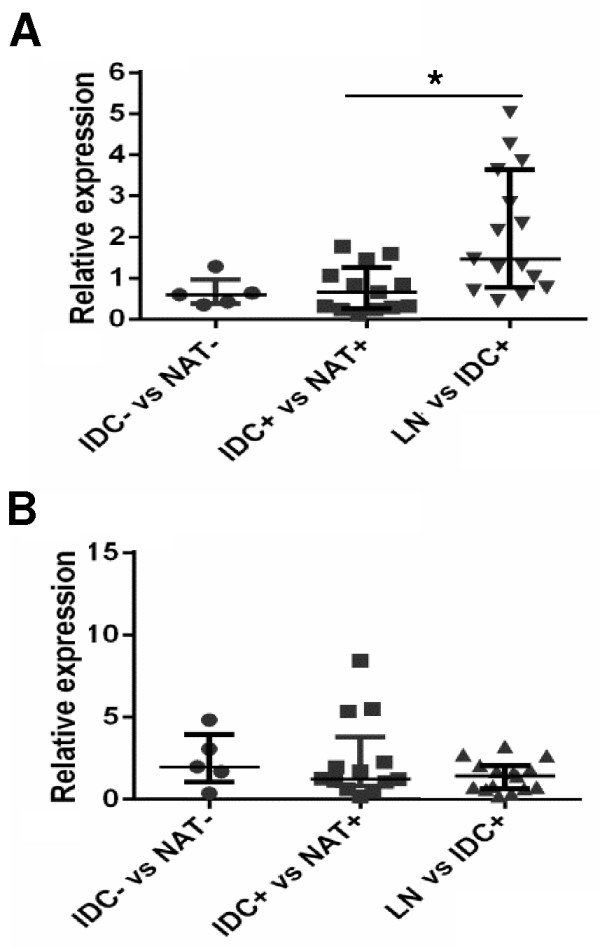
**Dicer and Drosha expression in tumours and lymph node metastases.** Relative quantification of **A)** Dicer and **B)** Drosha by real-time RT-PCR in NAT from LN- patients versus matched LN- tumours (IDC- vs NAT-, n = 5), NAT from LN + patients versus matched LN + tumours (IDC + vs NAT+, n = 13) and LN metastases versus matched LN + tumours (LN vs IDC+, n = 15). Results are shown as the fold change (2^-ΔΔCt^) of Dicer and Drosha expression in matched cases, *p = 0.0202.

There are mixed reports regarding whether Dicer and Drosha are associated with disease progression in breast cancer [18,19,21,22]. However, the relationship of these genes to progression and other clinical features in the TNBC subtype has not been studied. We examined the relative expression of Dicer and Drosha in matched LN metastases to determine if there was a progressive loss in Dicer or gain in Drosha from normal to tumour to metastasis. We compared this to the expression of these genes in tumours that were LN negative. The fold change in the expression of Dicer and Drosha in tumour compared to NAT was similar in LN + and LN- tumours (compare IDC- vs NAT- with IDC + vs NAT+, Figure 2). The fold increase in Drosha expression when LN metastases were compared to their matched primary tumour (LN vs IDC+) was not different to the increase observed in tumour versus NAT (IDC + vs NAT+) (Figure 2B). In contrast, there was a significant increase in Dicer expression when LN metastases were compared to their matched primary tumour (LN vs IDC+, p = 0.0202, Figure 2A). This suggests that while there is a decrease in Dicer in the progression from normal to tumour, there is a subsequent increase following metastases to the lymph node. The increase in Dicer expression in LN metastases was observed in 12/15 tumours analysed.

We examined whether the expression of Dicer or Drosha in tumours was associated with clinical features of breast cancer. We found no correlation of either Drosha or Dicer expression with age at diagnosis, tumour size or the number of positive lymph nodes (Table 1).

**Table 1:** Correlation between Dicer and Drosha expression levels with clinical variables in triple negative breast cancer

	**Dicer**	**Drosha**
**Age at diagnosis**		
*r*_s_^*^	-0.1756	-0.1240
*p*-*value*	0.3130	0.4778
**Tumour size**		
*r*_s_^*^	0.05331	0.1014
*p*-*value*	0.7610	0.5620
**No. of positive lymph nodes**		
*r*_s_^*^	0.08541	0.04540
*p*-*value*	0.6257	0.7957

*Values represent Spearman’s rank correlation coefficient.

## Discussion

Dicer and Drosha have been reported to be dysregulated in TNBC [21,23]. However, these studies used very few normal tissues in their analysis and the correlation with LN metastases was not examined. This study aimed to evaluate whether the relative expression of Dicer and Drosha was altered in TNBC and whether it was associated with clinical features and progression to LN metastases in this subtype.

Our results have shown that Drosha was expressed at significantly higher levels than Dicer in TNBC and that the expression of these two genes was highly correlated with one another, in agreement with the results of Passon *et al*. and Dedes *et al*. [21,23]. Decreased expression of Dicer has been noted in several cancers, while increased expression has been observed in ovarian, prostate and colorectal cancer [12-14]. We observed a significant decrease in Dicer expression in breast cancer when compared to matched NAT. The proportion of cases with decreased Dicer expression in this study (72%) is similar to that previously reported by Passon *et al*. (61.3%) and Dedes *et al*. (77.7%) [21,23]. In contrast, we observed a significant increase in Drosha expression in 78% of the tumour tissues analysed when compared to matched NAT. This confirms previous findings that Drosha is increased in TNBC [23]. The relevance of increased Drosha expression and decreased Dicer expression in TNBC is not known at present. It is possible that Dicer expression becomes decreased as a result of increased Drosha expression to limit miRNA biogenesis, thereby reducing miRNA function in the breast.

Reduced Dicer expression as well as over-expression has previously been reported to be associated with worse prognosis in breast and colorectal cancer respectively, indicating cancer specific differences in the prognostic value of this gene [14,19]. Although we did not have outcome data on the patients used in this study, we were able to examine whether Dicer and Drosha were differentially expressed in LN positive patients compared to LN negative patients and whether this expression was related to clinical features in the highly aggressive TNBC subtype. While Dicer was reduced in primary breast cancers of both LN positive and LN negative patients; unexpectedly, we found its expression significantly increased in LN metastases when compared to matched primary tumours. However, we saw no association of either Dicer or Drosha with clinical features including age at diagnosis, tumour size or the number of positive lymph nodes. This may be due to the small sample size used in this analysis, but TNBC represents a very specific proportion of all breast cancers and these specimens are difficult to obtain. This is the only study to date that has analysed Dicer and Drosha in TNBC samples compared to matched normal adjacent tissue and matched lymph node metastases.

A limitation of our study is that it was performed only on cDNA derived from FFPE tissues. Although FFPE tissues contain fragmented RNA, our real-time RT-PCR assays were designed with small amplicon sizes (Dicer- 65 bp, Drosha- 66 bp and β2-Microglobulin- 75 bp) to circumvent the requirement for intact RNA. Additionally, random hexamers were incorporated in the reverse-transcription procedure, which do not require the RNA to be intact, in contrast to reverse-transcription with oligo dT. Given the inconsistent correlations between these enzymes at the mRNA and protein level in the literature, and that the interpretation/quantification of Dicer and Drosha immunohistochemical staining is problematic, we did not perform immunohistochemical analyses on this cohort of tumours [21,22]. Although, the sample size used in this study is relatively small, the tumours are homogenous with regards to size, hormone receptor status and histological grade and this is the only study that has analysed matched TNBC cases and lymph node metastases.

## Conclusions

This study has shown that Dicer and Drosha are dysregulated in TNBC and matched LN metastases. We have shown that Dicer is down-regulated, while Drosha is up-regulated in primary breast cancers compared to NAT. There was no difference in Drosha expression in lymph node metastases when compared to the primary tumour, however, Dicer was significantly increased. This suggests that while there is a decrease in Dicer in the progression from NAT to tumour, there is a subsequent increase following metastases to the lymph node. The clinical relevance of this is under investigation.

## Abbreviations

TNBC: Triple negative breast cancer; miRNA: microRNA; RT-PCR: Reverse transcription polymerase chain reaction; ER: Estrogen receptor; PR: Progesterone receptor; HER2: Human epidermal growth factor receptor; LN: Lymph node; NAT: Normal adjacent tissue; IDC: Invasive ductal carcinoma; FFPE: Formalin fixed paraffin embedded; RNA: Ribonucleic acid; cDNA: complementary deoxyribonucleic acid.

## Competing interests

The authors declare they have no competing interests.

## Authors’ contributions

KAK: study concept and design, carried out experiments, analysis and interpretation of data, drafting of the manuscript. SGB: patient collection, material support, manuscript revision. JFF: study design, obtained funding, critical revision of the manuscript for important intellectual content. RJS: study design, obtained funding, critical revision of the manuscript for important intellectual content. All authors read and approved the final manuscript.

## Pre-publication history

The pre-publication history for this paper can be accessed here:

http://www.biomedcentral.com/1471-2407/14/253/prepub

## Supplementary Material

Additional file 1: Table S1 Average and median Cts for β2-Microglobulin in NAT, IDC and LNs. **Figure S1**. The expression of β2-Microglobulin in NAT, IDC and LNs. Values represent the median with interquartile ranges. No significant difference (p>0.05) was observed between the sub-groups (Kruskal-Wallis rank test followed by a Dunn’s Multiple Correction test). Click here for file
