# Supplementary material for: The expression of Dicer and Drosha in matched normal tissues, tumours and lymph node metastases in triple negative breast cancer
Source: BMC Cancer. 2014 Apr 11;14:253. doi: 10.1186/1471-2407-14-253 (PMC4021460; doi:10.1186/1471-2407-14-253)
Supplement: Additional file 1: Table S1 — Average and median Cts for β2-Microglobulin in NAT, IDC and LNs. Figure S1. The expression of β2-Microglobulin in NAT, IDC and LNs. Values represent the median with interquartile ranges. No significant difference (p>0.05) was observed between the sub-groups (Kruskal-Wallis rank test followed by a Dunn’s Multiple Correction test). [file 1471-2407-14-253-S1.pdf]

Table S1: Average and median Cts for β2-Microglobulin in NAT, IDC and LNs.

	Average Ct	Median Ct	SEM
NAT	23.91	23.71	0.29
IDC	22.92	23.08	0.46
LN	24.14	24.12	0.35

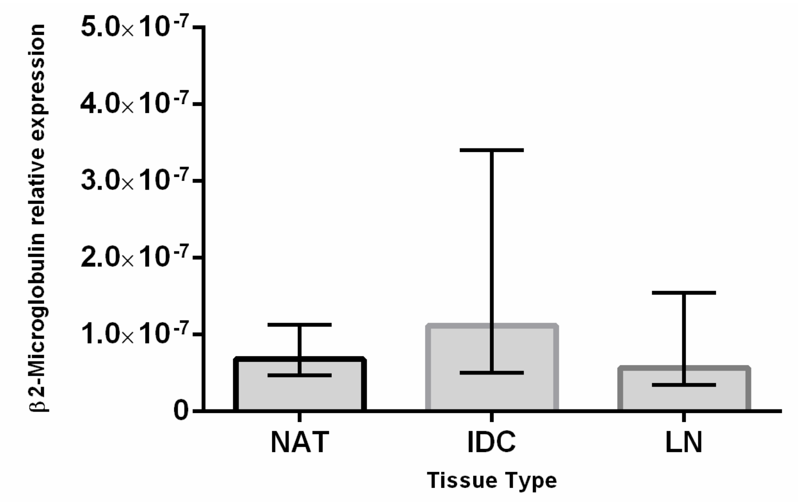

Figure S1: The expression of β2-Microglobulin in NAT, IDC and LNs. Values represent the median with interquartile ranges. No significant difference ($p>0.05$) was observed between the sub-groups (Kruskal-Wallis rank test followed by a Dunn's Multiple Correction test).
